# Commentary: Single-stranded telomere-binding protein employs a dual rheostat for binding affinity and specificity that drives function

**DOI:** 10.3389/fgene.2018.00742

**Published:** 2019-01-15

**Authors:** Ĺubomír Tomáška, Jozef Nosek, Regina Sepšiová, Filip Červenák, Katarína Juríková, Katarína Procházková, Martina Neboháčová, Smaranda Willcox, Jack D. Griffith

**Affiliations:** ^1^Department of Genetics, Faculty of Natural Sciences, Comenius University in Bratislava, Bratislava, Slovakia; ^2^Department of Biochemistry, Faculty of Natural Sciences, Comenius University in Bratislava, Bratislava, Slovakia; ^3^Lineberger Comprehensive Cancer Center, University of North Carolina, Chapel Hill, NC, United States

**Keywords:** evolution, yeast, telomere-binding protein, DNA-protein interaction, telomere

During evolution, protein-ligand interactions are continuously subjected to natural selection to generate their particular kinetic properties quantified in terms of binding affinities or specificities. Three decades ago (Kováč, 1987), it was argued that there is an upper limit for the specificity of interaction between binding partners (e.g., enzyme-substrate, ligand-receptor, protein-DNA sequence), since interactions that are too specific would lack flexibility, and a perfect recognition would be too rigid and possibly non-functional. This would imply that in some cases a decrease in specificity of binding might be beneficial for the host, especially when the more promiscuous binding might be helpful to keep up with an increased rate of evolutionary diversification of the ligands.

Telomeres, the nucleoprotein complexes at the ends of linear DNA chromosomes, are particularly useful for studying the evolution of DNA-protein interactions. With some exceptions, such as *Drosophila* (Kordyukova et al., 2018), the telomeric DNA consists of an array of short tandem repeats, where the double-stranded (ds) region is followed by a relatively short 3' single-stranded (ss) overhang. Both parts of telomeric DNA are bound by telomere-binding proteins (TBPs) constituting a platform for the formation of a protein complex called shelterin that plays essential roles in solving the end-replication and end-protection problems associated with the chromosomal termini (de Lange, 2018). Telomeric DNA repeats are very conserved, and in most eukaryotes, they are represented by the hexanucleotide 5′-TTAGGG-3′ or its variants (Blackburn, 2010). This is also the case for most basidiomycetous and ascomycetous fungi, such as *Ustilago maydis* (Guzmán and Sánchez, 1994) and *Neurospora crassa* (Schechtman, 1990), which harbor mammalian-type telomeric repeats. However, in hemiascomycetes, the telomeric repeats underwent a runaway evolution (Gunisova et al., 2009). In these fungi, the length, sequence and base composition of the repeats differ dramatically even between closely related species (Table 1). The situation is even more pronounced in species possessing heterogeneous telomeric repeats such as *Saccharomyces cerevisiae* and *Schizosaccharomyces pombe*. This poses a great challenge to TBPs, whose binding properties must rapidly co-evolve with their DNA targets (Steinberg-Neifach and Lue, 2015; Sepšiová et al., 2016; Červenák et al., 2017).

**Table 1:** Variability of telomeric repeats.

**Species**	Taxonomical classification^*^	**Telomeric repeat (5^**′**^ → 3^**′**^)**	**# nt**	**% [G/C]**
*Homo sapiens*	A/M	TTAGGG	6	50/0
*Arabidopsis thaliana*	P	TTTAGGG	7	43/0
*U. maydis*	F/B	TTAGGG	6	50/0
*N. crassa*	F/A/P	TTAGGG	6	50/0
*Y. lipolytica*	F/A/S	TTAGTCAGGG	10	40/10
*Spathaspora passalidarum*	F/A/S	TTCGGGGTACTCTCTTATGTTGCGGGTAGGATG	34	35/14
*C. albicans*	F/A/S	TctAactTctTGgtGTaCGGATG	23	26/17
*C. parapsilosis*	F/A/S	TtgAttaTacTGagGTcCGGATG	23	30/13
*S. cerevisiae*	F/A/S	TG_2−3_(TG)_1−6_	5-16	(max) 56/0
*S. pombe*	F/A/T	G_2−8_TTACAC_0−1_	7-14	(max) 57/14

*Representative examples that defy the rule that the telomeric repeat is conserved, short and G-rich are shown (reviewed in Červenák et al., 2017). Small letters in the corresponding sequences indicate variable positions in telomeric repeats of the closely related Candida species such as C. albicans and C. parapsilosis illustrating a rapid evolution of the repeats in yeasts*.

* *A/M, Animalia/Mammalia; P, Plantae, F/B, Fungi/Basidiomycota; F/A/P, Fungi/Ascomycota/Pezizomycotina; F/A/S, Fungi/Ascomycota/Saccharomycotina; F/A/T, Fungi/Ascomycota/Taphrinomycotina*.

Over the past few years this question has been addressed through biochemical and genetic analysis of TBPs from various yeast species (Kramara et al., 2010; Lue, 2010; Visacka et al., 2012; Steinberg-Neifach and Lue, 2015; Sepšiová et al., 2016; Červenák et al., 2017). Our studies have shown that Tay1, the dsTBP of the yeast *Yarrowia lipolytica* (Kramara et al., 2010) exhibits lower affinity for its own telomeres than for the mammalian-type telomeric repeats (Table 1). This led us to the conclusion that “the binding properties of a DNA-binding protein are tuned to inferior values, thus enabling its dynamic association with the target DNA loci” (Visacka et al., 2012). This is in line with the conclusions that enzymes usually exhibit kinetic parameters much below the diffusion limit, and thus, that evolution does not prefer kinetically superior catalysts (Bar-Even et al., 2011).

Moreover, in line with Kováč, we have argued that the high specificity of the ancestral dsTBP at some point of evolution became a burden for performing its telomeric functions, posing a selection pressure for its replacement by a less specific DNA-binding protein (Sepšiová et al., 2016; Červenák et al., 2017). For example, in *S. pombe*, the Tay1 homolog (Teb1) was replaced by a more flexible and less specific Taz1 protein that is able to bind a wide range of telomeric repeat variants (Vassetzky et al., 1999; Sepšiová et al., 2016). Similar situations occurred in *S. cerevisiae* and closely related species, where the function of the principal dsTBP is fulfilled by Rap1, a protein exhibiting flexible DNA-binding properties (Piña et al., 2003; Steinberg-Neifach and Lue, 2015). In contrast, when the telomeres of *S. cerevisiae* are artificially “humanized,” Rap1 is no longer able to fulfill its telomeric function, and a more specific protein (e.g., Tbf1) is recruited to the chromosomal ends (Brevet et al., 2003). These results imply that fine-tuning of DNA-binding specificity (in either direction) accompanied co-evolution of TBPs with telomeric repeats in ascomycetous yeasts.

Recently, Glustrom et al. performed an elegant systematic analysis of the effects of amino acid substitutions across the ssTBP of *S. cerevisiae* (Cdc13p) DNA-binding interface on its affinity and specificity toward heterogeneous telomeric repeats of the host cell (Glustrom et al., 2018). The authors showed that, as they expected, a subset of mutants exhibiting a significant loss in affinity *in vitro* also conferred a profound loss of viability *in vivo*. To the authors' surprise the mutant proteins with an increased specificity conferred “a gradient of viability *in vivo* that paralleled the loss in sequence tolerance *in vitro*, arguing that binding specificity can be fine-tuned to ensure optimal function.” They conclude “while it is common to observe loss of function upon loss of a biochemical activity, the enhancement of specificity leading to a substantial reduction in biological function has not been reported previously in nucleic acid recognition, to the best of our knowledge.”

We appreciate the elegance of the study and agree that “binding specificity is fine-tuned to ensure optimal function.” However, we do not share the authors' conclusion that reduction in biological function has not been reported previously in nucleic acid recognition. The examples mentioned above demonstrate that during the evolution of telomeres in the ascomycetous fungi, bi-directional changes in specificity of DNA-binding proteins have been encountered numerous times. An extreme example is a mitochondrial telomere-binding protein (mtTBP) of *Candida parapsilosis*. Its low binding specificity enables it to provide two crucial functions *in vivo*—to protect the single-stranded telomeric overhang of linear mitochondrial DNA and to function as a non-specific single-stranded DNA-binding (SSB) protein involved in the replication of the mitochondrial DNA (Tomáška et al., 1997, 2001; Nosek et al., 1999).

Indeed, the idea that in the matter of DNA-binding the stronger and more specific may not be “functionally” the better is not limited to telomeres but has also been observed in the case of other DNA-binding proteins. For example, particular mutations that increase the binding affinity of herpes simplex virus processivity factor UL42 to DNA result in reduced DNA replication fidelity (Jiang et al., 2009). Furthermore, studies investigating the evolution of transcription factors indicate that decreasing specificity accompanied by an increase in flexibility of DNA-binding depends on an evolutionary context (e.g., Ramos and Barolo, 2013; McKeown et al., 2014).

In summary, the results of Glustrom et al. (2018) nicely complement the conclusions inferred from previously published studies on the puzzling diversity of yeast telomeres as well as on the evolution of DNA-binding proteins. In more general terms, they also provide additional evidence that increasing perfection of molecular recognition does not necessarily mean an optimal evolutionary strategy (Kováč, 1987; Bar-Even et al., 2011).

## Author Contributions

All authors listed have made a substantial, direct and intellectual contribution to the work, and approved it for publication.

### Conflict of Interest Statement

The authors declare that the research was conducted in the absence of any commercial or financial relationships that could be construed as a potential conflict of interest.
